# Human Regulatory Dendritic Cells Develop From Monocytes in Response to Signals From Regulatory and Helper T Cells

**DOI:** 10.3389/fimmu.2020.01982

**Published:** 2020-08-18

**Authors:** Xiangyue Zhang, Pingping Zheng, Tyler R. Prestwood, Hong Zhang, Yaron Carmi, Lorna L. Tolentino, Nancy Wu, Okmi Choi, Daniel A. Winer, Samuel Strober, Eun-Suk Kang, Michael N. Alonso, Edgar G. Engleman

**Affiliations:** ^1^Department of Pathology, Blood Center, Stanford University School of Medicine, Palo Alto, CA, United States; ^2^Bone Marrow Transplantation, Stanford University School of Medicine, Palo Alto, CA, United States; ^3^Buck Institute for Research on Aging, Novato, CA, United States; ^4^Division of Cellular and Molecular Biology, Diabetes Research Group, Toronto General Hospital Research Institute (TGHRI), University Health Network, Toronto, ON, Canada; ^5^Division of Immunology and Rheumatology, Department of Medicine, Stanford University of Medicine, Stanford, CA, United States; ^6^Department of Laboratory Medicine and Genetics, Samsung Medical Center, Sungkyunkwan University, Seoul, South Korea

**Keywords:** DCs, DC_*Reg*_, Tregs, Th, monocytes

## Abstract

Dendritic cells (DCs) are powerful antigen presenting cells, derived from bone marrow progenitors (cDCs) and monocytes (moDCs), that can shape the immune response by priming either proinflammatory or tolerogenic immune effector cells. The cellular mechanisms responsible for the generation of DCs that will prime a proinflammatory or tolerogenic response are poorly understood. Here we describe a novel mechanism by which tolerogenic DCs are formed from monocytes. When human monocytes were cultured with CD4^+^FoxP3^+^ natural regulatory T cells (Tregs) and T helper cells (Th) from healthy donor blood, they differentiated into regulatory DCs (DC_*Reg*_), capable of generating induced Tregs from naïve T cells. DC_*Reg*_ exhibited morphology, surface phenotype, cytokine secretion, and transcriptome that were distinct from other moDCs including those derived from monocytes cultured with Th or with GM-CSF/IL-4, as well as macrophages (MΦ). Direct cell contact between monocytes, Tregs and Th, along with Treg-derived CTLA-4, IL-10 and TGF-β, was required for the phenotypic differentiation of DC_*Reg*_, although only IL-10 was required for imprinting the Treg-inducing capacity of DC_*Reg*_. High ratios of Treg:Th, along with monocytes and DC_*Reg*_ similar in function and phenotype to those induced *in vitro*, were present *in situ* in human colorectal cancer specimens. Thus, through the combined actions of Tregs and Th, monocytes differentiate into DCs with regulatory properties, forming a positive feedback loop to reinforce Treg initiated immune regulation. This mechanism may contribute to immune tolerance in tissues such as tumors, which contain an abundance of Tregs, Th and monocytes.

## Introduction

As one component of the “mononuclear phagocyte system” (MPS), monocytes constitute approximately 10% and 4% of the leukocytes in human and murine peripheral blood, respectively (1). Circulating monocytes can infiltrate into mucosal (2), inflammatory (3), cancer tissues (4), or draining lymph nodes (LNs) (5) and differentiate into either macrophages (MΦ) or dendritic cells (moDCs) (6). Monocytes are highly plastic and their differentiation is subject to the signals that they receive (7), which enables the cells to acquire distinctive features to promote or hamper immune responses (1). We previously observed that human CD4^+^ Th cells and monocytes frequently interact with one another in inflamed tissues of patients with autoimmune and allergic disease, and demonstrated that such interactions result in the differentiation of monocytes into pro-inflammatory immunogenic moDCs (DC_*Th*_), which in turn induce the formation of Th effector cells from naïve T cells (8, 9). The differentiation of DC_*Th*_ from monocytes occurs in a cell contact, GM-CSF and TNFα dependent manner (8).

Although numerous inflammatory moDC subsets have been identified in inflammatory environments (8–11), little is known about the signals required to induce tolerogenic/immunoregulatory moDCs. Since regulatory T cells (Tregs) play a central role in promoting immune tolerance and maintaining immune homeostasis (10), we and others considered the possibility that they might directly induce regulatory moDCs (DC_*Reg*_) from monocytes in a manner analogous to the induction of DC_*Th*_ by Th cells. However, when monocytes are cultured with activated Tregs alone, they become macrophages (MΦ) (12). Since Tregs most often exert direct immune suppression on Th cells (11, 13, 14), we hypothesized that both Tregs and Th might be required for the generation of immune regulatory DCs from monocytes. To evaluate this hypothesis, we cultured classical human CD14^+^ monocytes with activated natural Tregs and Th from healthy donors. The results show that under these conditions monocytes differentiate into regulatory DC_*Reg*_ with the capacity to induce the formation of immune suppressive CD4^+^FoxP3^+^ Tregs. DC_*Reg*_ are distinctive in their morphology, phenotype, cytokine secretion, and transcriptome. DC_*Reg*_ similar in phenotype and function to those induced *in vitro* were present *in situ* in colorectal cancer (CRC), along with an abundance of monocytes, Tregs and Th cells. Therefore, our study reveals a novel mechanism by which Tregs can inhibit the immune response by inducing the generation of DC_*R*__*eg*_.

## Materials and Methods

### Processing of Human PBMCs

PBMCs were isolated from buffy coats/LRS chambers obtained from healthy adult blood donors at the Stanford Blood Center. CD14^+^ monocytes and CD4^+^CD127^*low*^ T cells were isolated from PBMCs using RosetteSep Human Monocyte Enrichment Kits and CD4^+^CD127^*low*^ T cell Enrichment Kits, respectively (Stem Cell Technologies). CD14^+^ monocytes were further purified to > 97% purity by magnetic separation with anti-CD14 conjugated microbeads (Miltenyi Biotec). Memory CD4^+^ Th and Tregs were obtained by sorting pre-enriched T cells with fluorescently labeled mAbs against CD4, CD25, CD127, CD45RA, CD45RO and lineage markers along with propidium iodide (PI; Life Technologies). Th were defined as PI^–^Lin^–^CD4^+^CD45RA^–^CD45RO^+^CD127^+^CD25^–/low^, and Tregs were defined as PI^–^Lin^–^CD4^+^CD127^*low*^ CD25^+^. Cells were sorted with a BD FACSAria II^TM^ after they had been stained with mAbs to CD25 (BC96), CD14 (HCD14), CD19 (H1B19), CD20 (2H7) and CD56 (HCD56) (BioLegend) and CD4 (S3.5), CD45RA (MEM-56), CD45RO (UCHL1) (Life Technologies).

### Human Monocyte/T-Cell Cocultures

Monocytes (1 × 10^6^) were cultured with allogeneic Th or Tregs at a 10:1 ratio or Th and Tregs at 10:1:1 in 12-well plates (Corning) containing IMDM medium (Gibco) supplemented with 10% human serum, 2% FCS, 100U/ml penicillin, 100 μg/ml streptomycin, 2mM L-glutamine, sodium pyruvate, non-essential amino acids, 50 μM 2-ME, 50 ng/ml anti-CD3 (OKT3, BioLegend) and, where indicated, recombinant human IL-2 (Peprotech). GM-CSF and IL-4 derived DCs (DC_*GM*_) were generated as described (11). For imaging studies, cells were evaluated on day 4 by Leica DMIRB microscopy with a 40 × /0.55 Hoffman Modulation Contrast objective, and images were acquired using a digital ORCA-ER camera and Openlab acquisition software. Aliquots of cells were processed on day 4 with 5mM EDTA and subsequently stained with DAPI (Life Technologies) or LIVE/DEAD Fixable Aqua Dead Cell Stain Kit (Life Technologies), fluorescently labeled isotype control mAbs, or specific mAbs against CD3 (OKT3), CD2 (TS1/8), CD25 (BC96), CD45 (HI30), CD11c (3.9), CD163 (GHI/61), CD14 (61D3), CD40 (G28.5), CD80 (2D10), CD86 (IT2.2), CD274 (29E.2A3) from BioLegend; and/or HLA-DR (G46-6), CD209 (DCN46) from BD Pharmingen; and/or CD45RA (MEM-56), CD45RO (UCHL1) from Life Technologies; and/or CD127 (eBioRDR5) from eBioscience. Other cell aliquots were stimulated on day 3 with 1 μg/ml LPS for 16 h and stained with the above mAbs. Cells were analyzed on a BD LSRII flow cytometer, and FACS plots and MFIs were generated by FlowJo (Treestar). T cells were excluded according to their FSC:SSC profile and CD3/CD2 expression. For neutralizing studies, isotype control mAbs or neutralizing mAbs against CTLA-4 (5 μg/ml; BioLegend), IL-10 (2 μg/ml) or TGFβ (2 μg/ml; R&D Systems) were added at the initiation of culture. Transwell experiments were performed using 12/24-well 0.4 μM transwell inserts (Corning) under the indicated conditions. Flow cytometry analysis and FACS sorting were performed at Stanford Blood Center Flow Cytometry Lab.

### Mixed Leukocyte Reaction (MLR)

CD14^+^ monocytes were cultured as indicated earlier and stimulated with 1 μg/ml LPS on day 3. After 18 h, cells were washed 3x in PBS and HLA-DR^+^CD2^–^ DCs were purified by FACS and incubated with allogeneic naïve CD4^+^ T cells (10^5^/well) at a DC to T cell ratio of 1:2 in the presence or absence of 2 μg/ml anti-TGFβ. The CD4^+^ T cells for these assays were purified from PBMCs using a RosetteSep CD4^+^ Human T cell Isolation Kit (Stem Cell Technologies) followed by magnetic purification (>95% CD2^+^CD4^+^CD45RA^+^ cells by flow cytometry) using a Naïve CD4^+^ T Cell Isolation Kit II (Miltenyi Biotec) and subsequently labeled with CFSE. After 6 days, responder CD4^+^ T cells were analyzed for CD25 and FoxP3 expression. CD2^+^CD4^+^CD25^+^CFSE^–^ cells were further isolated by FACS and cocultured with 10^5^ CFSE labeled allogeneic CD45RA^+^CD4^+^ naïve responder T cells in a new MLR with irradiated autologous DCs (5 × 10^4^), which had been purified with human CD11c microbeads (Miltenyi) and anti-CD3 (0.5 ng/ml, plate-bound).

### Luminex Assays

Human Luminex 63-plex assays were performed by the Human Immune Monitoring Core at the Stanford Institute for Immunity, Transplantation and Infection. Samples were run in duplicate, and data were analyzed with GraphPad Prism6 (GraphPad-PRISM, Inc). Error bars represent SEM. Statistical differences for the mean values are indicated as follows: ^∗^*P* < 0.05; ^∗∗^*P* < 0.001; ^∗∗∗^*P* < 0.0001; ns, not significant.

### Microarray Analysis

DC_*Th*_, DC_*Reg*_, MΦ_*Treg*_ and DC_*GM*_ were sorted on a FACSAria (BD) and RNA was extracted with an RNeasy Micro Kit (Qiagen). Total RNA samples were sent to Stanford Functional Genomics Facility and microarray was performed on GeneChip Human Gene 2.0 ST Array from Affymetrix. Microarray data were analyzed using the *oligo* (13) and annotated with data base *hugene20sttranscriptcluster.db* (14) in Bioconductor. *limma* package (15) was applied to identify the differentially expressed genes among cell populations. *P*-values were adjusted with false discovery rate (FDR) and genes with FDR adjusted *P* value < 0.05 were selected to be differentially expressed genes. Principal component analysis on the differentially expressed genes was performed using *prcomp* in R and plotted with *ggplot2* package (version 2.2.1) (16). Heatmaps were generated using unsupervised clustering in the *pheatmap* package (version 1.0.8) (17). Biological functional gene ontology analysis was done with topGO (18). All the above data analyses were performed in R (version R 3.3.2^1^). Pairwise gene set enrichment analyses (GSEA) were applied for assessment of the similarity of DC_*Th*_, DC_*Reg*_, MΦ_*Treg*_ and DC_*GM*_ with the well-defined reference gene signatures by pairwise transcriptomes comparison. GSEA was done using Bubble Map module of Bubble GUM version 1.3.19 (19). The cell-specific gene fingerprints from previous published reports were selected as references: six gene sets of human DC subsets (DC1, DC2, DC3, DC4, DC5, DC6) from Science (20); one gene set of Macrophage reference gene signature generated by Segura et al. (21); and another gene set of VITD3 DCs (22). The BubbleMap returned a bubble map of pairwise GSEA results to show enrichment of a given gene set (reference) in a pairwise comparison. The normalized enrichment score (NES) and corrected *P*-value (FDR) of each bubble were also generated (19). Enhanced Volcano and ggplot2 were used for volcano plots using gene expression and adjusted *p* value.

### Processing of Human Colon Cancer Samples

Fresh CRC tissues were obtained from the Stanford Tissue Bank in accordance with IRB protocol 6304 following surgical resection of primary tumors. Some of the tissues were minced with surgical scissors and transferred to 15ml scintillation vials containing 2 mg/ml collagenase type IV (Worthington Biochemical Corporation) and 50 U/ml DNase I (Roche) for 30min at 37°C with constant agitation. Samples were subsequently filtered through a 70 μm filter and resuspended in PBS containing 2% human serum and 2mM EDTA. CD4^+^ T cells were isolated from the cell suspension with CD4 MicroBeads (Miltenyi Biotec) and further purified by FACS. DCs were FACS-sorted from the CD4 negative population. Samples of the same tissues were sectioned, stained and analyzed as described. The primary antibodies used were rabbit polyclonal anti-CD14 (1:200; Atlas), mouse monoclonal anti-FoxP3 (1:100) and rabbit anti-mouse T-bet (1:100; Santa Cruz Biotechnology).

Human blood collection to obtain PBMCs and CRC collection were approved by the Stanford Research Compliance Office and performed according to institutional guidelines under Stanford IRB protocol 6304, “Dendritic and T cell Signaling in Gastrointestinal Cancer.” All sequencing data are publicly available from NCBI’s Gene Expression Omnibus at GEO accession GSE148114.

See Supplemental Experimental Procedures for transmission electron microscopy, endocytosis and phagocytosis, CFSE labeling, and RNA isolation and quantitative RT-PCR.

## Results

### In the Presence of Both Tregs and Th, Human Monocytes Differentiate Into Regulatory DCs That Induce CD4^+^ FoxP3^+^ Tregs

**FIGURE 1:**
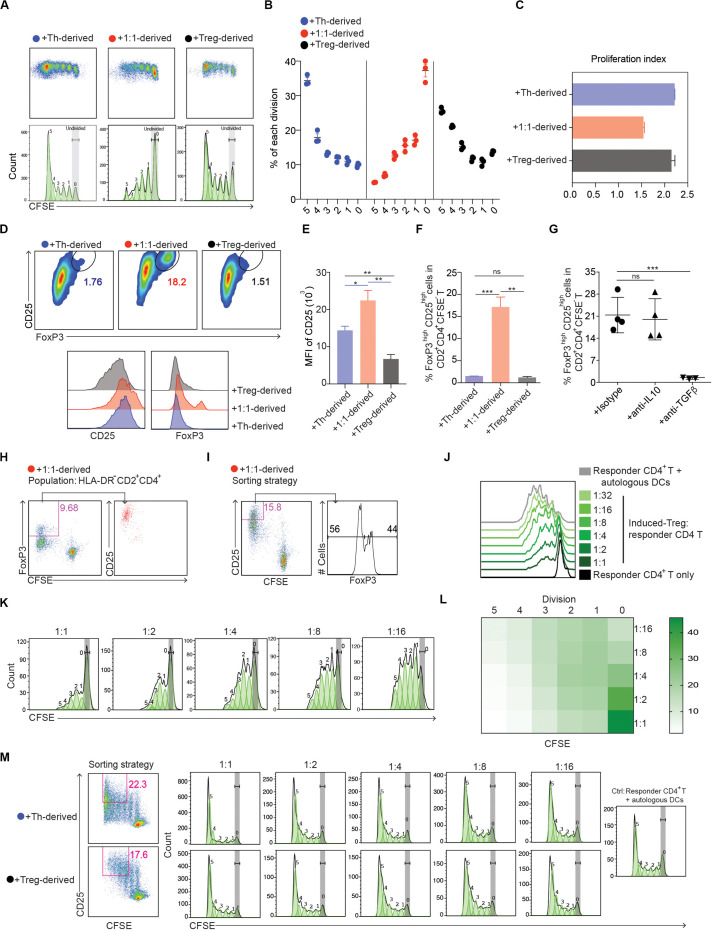
Human monocytes cultured with defined mixtures of Tregs and Th differentiate into cells that induce immune suppressive CD4^+^FoxP3^+^ Tregs. **(A)** Dotplots and histograms showing CFSE dilution of CD4^+^ T cells cultured with monocyte-derived cells from the CD4^+^ T cell-monocyte cultures. **(B)** Graphical representation of statistical analysis of CFSE^–^ cell percentage (mean ± SEM, *n* = 3) obtained from **(A)**. Similar results were obtained in two additional experiments. **(C)** Proliferation index calculated by Flowjo. **(D)** Expression of CD25 vs. FoxP3 in CFSE^–^CD2^+^CD4^+^ cells. Numbers adjacent to gated areas indicate percentage of gated cells. **(E,F)** Graphical representation of statistical analysis of the MFI of CD25 and the percentage of CD25^*high*^FoxP3^*high*^ cells in CD2^+^CD4^+^CFSE^–^ proliferated T cells. **(G)** CD14^+^ monocytes were cultured with Tregs and Th at a ratio of 10:1:1 in the presence of anti-IL-10, 2 μg/ml; anti-TGFβ, 2 μg/ml or isotype control antibody, 5 μg/ml. After 72 h, 1 μg/ml LPS was added, and 16 h later FACS-purified myeloid cells (DR^+^CD2^–^) were further cultured with MACS sorted CFSE-labeled naïve allogeneic CD4^+^ T cells. The percentage of FoxP3^*high*^ cells in CD2^+^CD4^+^CFSE^–^ cells was determined 6 days later. Mean ± SEM, *n* = 5. Similar results were obtained in two additional experiments. *P < 0.05; **P < 0.001; ***P < 0.0001; ns, not significant. Data were analyzed with *one-way* ANOVA, followed by Dunnett’s test for multiple comparisons. **(H,I)** 10^5^ CFSE labeled CD45RA^+^CD4^+^ T cells were cultured with 5 × 10^4^ allogeneic myeloid cells purified from 3-day cultures of monocytes, Tregs and Th at 10:1:1. After 6 days the cells were analyzed by flow cytometry. Responder CD4^+^ T cells were gated as CD2^+^CD4^+^. **(H)** CFSE^–^FoxP3^+^ DC_*Reg*_-induced Tregs were analyzed for CD25 expression. **(I)** CD25 expression was used as the basis for sorting CD2^+^CD4^+^CFSE^–^ cells. Percentages of FoxP3^–^ and FoxP3^+^ cells in CD2^+^CD4^+^CFSE^–^CD25^+^ cells are shown. **(J–M)** CD2^+^CD4^+^CFSE^–^CD25^+^ cells induced by 1:1 (Th:Treg), Th or Treg-derived myeloid cells were purified by FACS and cultured in a new MLR containing 10^5^ CFSE labeled allogeneic CD45RA^+^CD4^+^ cells and irradiated DCs (5 × 10^4^) and anti-CD3 (0.5 ng/ml, plate-bound). After 84–90 h, proliferation of CFSE-labeled naïve responder CD4^+^ T cells was analyzed by flow cytometry. Cells were gated as DAPI^–^CD2^+^CD4^+^. Data are representative of 2 independent experiments. **(J–L)** Dose-dependent suppression of induced Tregs on responder CD4^+^ T cells. **(L)** Heatmap of statistical analysis of the responder CD4^+^ T cell proliferation as indicated by CFSE in each division defined by Flowjo. Mean ± SEM, *n* = 3. Similar results were obtained in both experiments.

To test our hypothesis, freshly isolated CD14^+^ monocytes from the blood of healthy donors were cultured with Th alone, Tregs alone or with Tregs and Th cells at a ratio of 1:1. Prior to culture, each population had been sorted to > 99% purity (Supplementary Figure S1A) and the Tregs shown to suppress Th proliferation (Supplementary Figure S1B). After 3 days of culture, the myeloid cells (HLA-DR^+^CD2^–^) were sorted (>99% purity), activated with LPS and incubated for 6 days with CFSE-labeled naïve CD4^+^ T cells. While monocytes that had been cultured with Tregs and Th at 1:1 elicited weaker CD4^+^ T cell proliferative responses (Figures 1A–C) compared with those cultured with Th or Tregs alone, a significant proportion of T cells that proliferated developed into CD25^*high*^FoxP3^*high*^ Tregs (Figures 1D–F). In contrast, monocytes cultured with Th or Tregs alone failed to induce CD25^*high*^FoxP3^*high*^ Tregs (Figures 1D–F), although Th cultured monocytes induced vigorous CD4^+^ T cell proliferation and CD25 expression. Treg differentiation was dependent on TGFβ, but not IL-10, since addition of neutralizing anti-TGFβ antibody, but not neutralizing anti-IL-10 antibody, during the MLR abrogated FoxP3 expression by proliferating T cells (Figure 1G).

To confirm that the FoxP3^+^ T cells induced from monocytes cultured with Th and Tregs at 1:1 were immunosuppressive, we FACS purified CD25^*high*^CFSE^–^ T cells (∼50% FoxP3^+^) (Figures 1H,I) and tested their ability to inhibit the activation of naïve CD4^+^ T cells (Supplementary Figure S2) stimulated with autologous DCs and anti-CD3 mAb. The purified CD25^*high*^CFSE^–^CD4^+^ T cells suppressed naïve T cell proliferation in a dose-dependent manner (Figures 1J–L), whereas purified CD25^+^CFSE^–^CD4^+^ T cells obtained from the MLRs containing Th or Tregs alone had no suppressive effect (Figure 1M). Collectively, these data demonstrate that in the presence of Th, Tregs from healthy donors promote the formation of CD4^+^FoxP3^+^ Treg-inducing myeloid cells from monocytes.

### Regulatory DCs Derived From Monocytes Following Their Culture With Tregs and Th Are Phenotypically and Functionally Distinct From DC_*Th*_ and MΦ_*Treg*_

**FIGURE 2:**
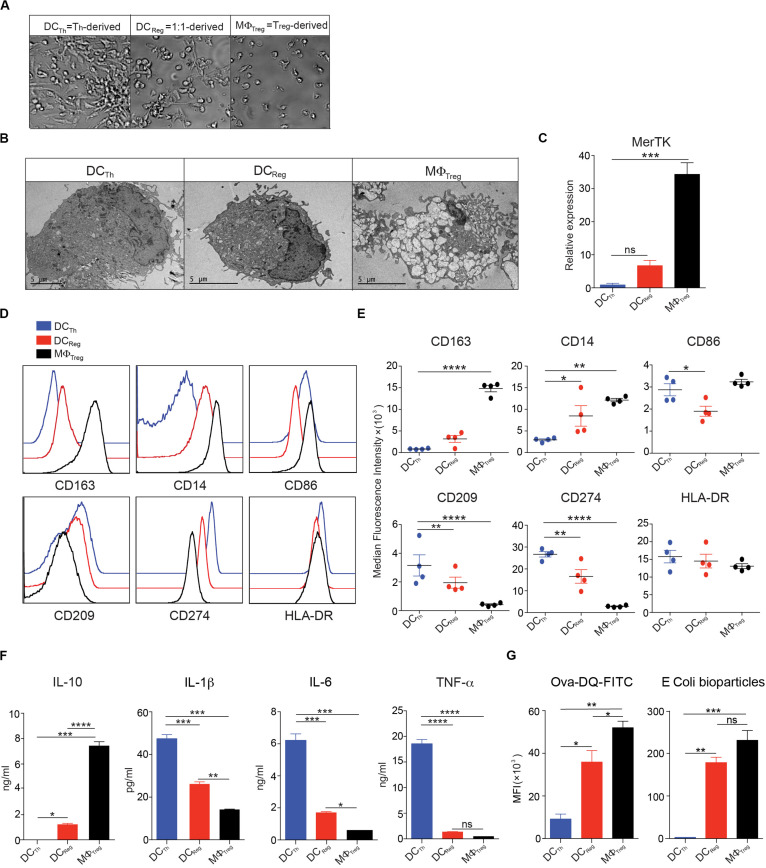
Monocytes cultured with Tregs and Th differentiate into DC-like cells with unique morphology and surface phenotype. **(A–G)** In the presence of anti-CD3 mAb, CD14^+^ monocytes were cultured with Tregs (10:1) or memory Th (10:1) alone, or with Tregs and Th at a 10:1:1 ratio. **(A)** Images were captured with a bright-field DIC microscope on day 4 of culture; original magnification 100X. **(B)** Transmission electron micrographs of FACS sorted HLA-DR^+^CD2^–^ cells on day 4 of culture. **(C)** MerTK in FACS purified HLA-DR^+^CD2^–^ cells assayed by real-time PCR on day 4 of culture. **(D,E)** Flow cytometric analysis of monocyte-derived HLA-DR^+^CD2^–^ cells on day 4 of culture. **(F)** On day 4, HLA-DR^+^CD2^–^ cells were purified by FACS, and 200,000 cells were incubated in 200 μl culture medium containing 1 μg/ml LPS. After 16h, cell-free supernatants were assayed by Luminex, and IL-10, IL-1β, IL-6, and TNFα levels are shown. **(G)** Left: On day 3, 1 μg/ml LPS was added and 16h later cells harvested from the cocultures were incubated with OVA-DQ for 60 min at 37°, and HLA-DR^+^CD2^–^ cells were analyzed for processing of OVA. MFIs of green signal are shown. Right: Cells harvested from the cocultures were incubated with PE-conjugated *E. coli* bioparticles for 60 min at 37°C. MFIs of PE in HLA-DR^+^CD2^–^ cells are shown. Mean ± SEM of 2 independent experiments and > 8 donors. Data are representative of at least 3 independent experiments. *P < 0.05, **P < 0.01, ***P < 0.001, ****P < 0.0001; ns, not significant. Data were analyzed with *one-way* ANOVA, followed by Dunnett’s test for multiple comparisons.

Given the stark functional differences between the cells derived from monocytes cultured with Th alone and those cultured with mixtures of Th and Tregs, we decided to study these populations in greater detail. Consistent with a previous study (8), monocytes that had been cultured with Th alone developed prominent dendrites within 24h and these DC-like changes peaked at day 4; hence, we refer to these cells as DC_*Th*_. The addition of Tregs to fresh cultures of monocytes and Th, at a ratio of Th:Treg of 1:1, resulted in the formation of cells with fewer and shorter dendrites that were otherwise similar in appearance to the DC_*Th*_. Based on their DC-like appearance and FoxP3^+^ Treg-inducing capacity (Figures 1A–F), we refer to these cells as DC_*Reg*_ (Figure 2A). Examination of the cells by electron microscopy confirmed that both DC_*T*__*h*_ and DC_*Reg*_ had typical DC morphology with large nuclei, dense cytoplasm and numerous dendrites. In contrast, monocytes cultured with Tregs in the absence of Th developed numerous intracellular vesicles and a small nucleus (Figure 2B), characteristic of classical MΦ, and we refer to these cells as MΦ_*Treg*_.

We next analyzed the molecular phenotype of each cell population as an additional means to assess the differences and similarities between DC_*Reg*_, DC_*Th*_ and MΦ_*Treg*_. As expected, MΦ_*Treg*_ expressed high transcript levels of the MΦ-specific receptor MerTK (23–25) (Figure 2C) and high levels of CD14 and CD163 proteins. In contrast, DC_*Th*_ expressed low levels of these molecules (Figures 2D,E). DC_*Reg*_ exhibited low expression of MerTK and CD163 and intermediate expression of CD14 and CD209 (Figures 2D,E).

**FIGURE 3:**
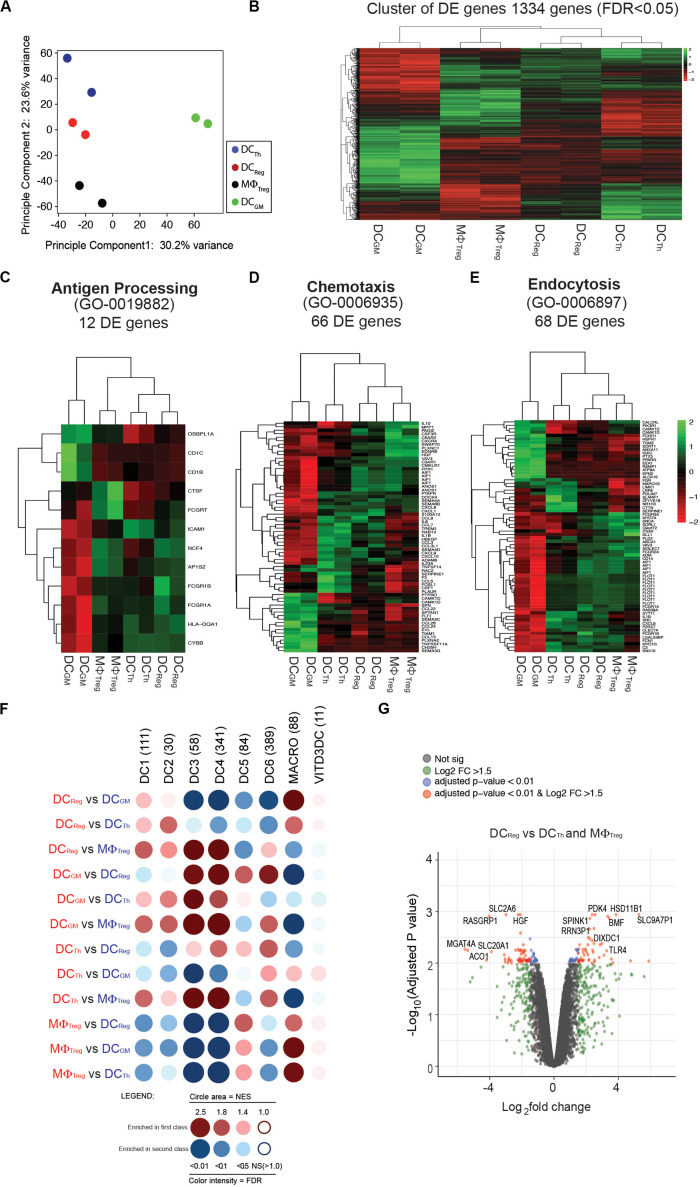
Transcriptomic profile of DC_*Reg*_ further distinguishes these cells from DC_*Th*_, MΦ_*Treg*_ and DC_*GM*_. **(A)** Principal component analysis (PCA) for DC_*Th*_, DC_*Reg*_, MΦ_*Treg*_ and DC_*GM*_ from each sample based on the mean expression of the genes with variable expression. **(B)** Cluster analysis of a total of 1334 differentially expressed genes [false discovery rate (FDR) < 0.05]. **(C)** Hierarchical clustering using sets of differentially expressed genes corresponding to the gene ontology biological functions of antigen processing (53 genes), **(D)** chemotaxis (73 genes), or **(E)** endocytosis (100 genes). **(F)** GSEA of the gene signature (GeneSet) of DC_*Th*_, DC_*Reg*_, MΦ_*Treg*_ and DC_*GM.*_ GSEA results for pairwise comparisons between human blood CD141^+^Clec9A^+^ (labeled as DC1), CD1c_A (DC2), CD1c_B (DC3), CD141^–^CD1c^–^ DCs (DC4), pDCs (DC6), DC5/AS DCs (DC5), macrophage (MACRO), and VITD3DC gene sets. Dot blot representation of all pairwise GSEA comparisons. GeneSets comprise different number of genes (n). Dot color corresponds to the font color of the population in which the GeneSet is enriched. The dot area is proportional to the NES (Normalized enrichment score) which varies from 1 (no enrichment) to a maximum of 5. The color intensity is indicative of the FDR statistical *q* value, which estimates the likelihood that the enrichment of the GeneSet represents a false-positive finding. **(G)** Volcano plot representation of microarray data. DC_*Reg*_ vs. DC_*Th*_ and MΦ_*T*__*reg*_ were plotted according to the log_2_ fold change (*X* axis) and log_10_ adjusted *p*-value (*Y* axis). Log2FC represents the absolute value of log_2_ fold change. Log2FC and *p* value was calculated with the Benjamini-Hochberg (BH) method.

**FIGURE 4:**
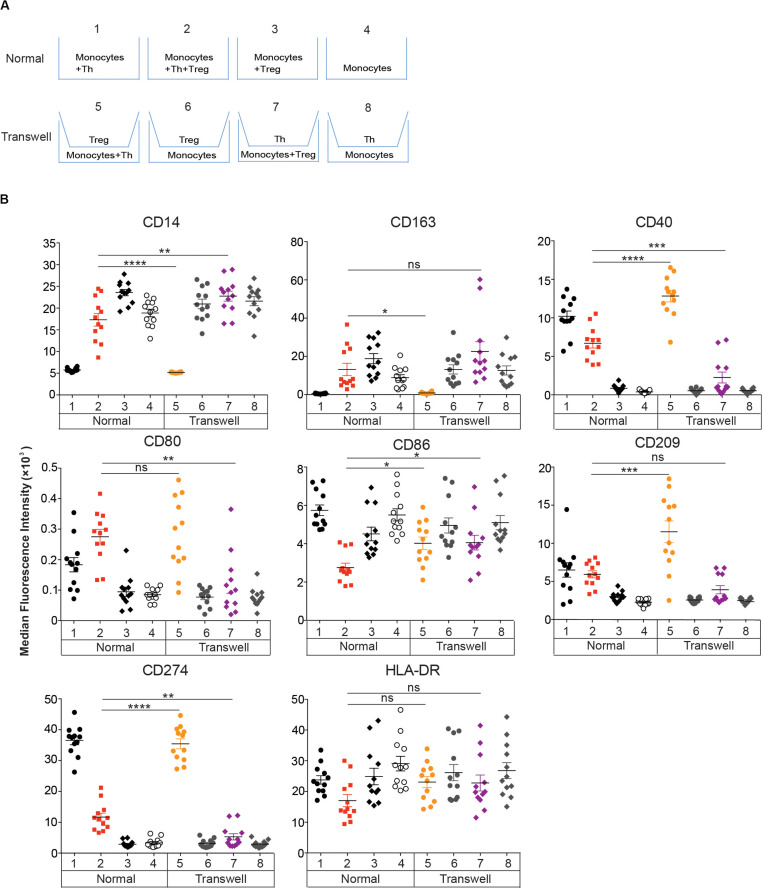
Effect of Tregs on DC_*Reg*_ differentiation requires cell-cell contact. **(A,B)** CD14^+^ monocytes were cultured for 4 days with Tregs or memory Th alone, or with Tregs and Th together at a 1:1 ratio as in Figure 2. **(A)** Standard and transwell cocultures were performed as described in section “Materials and Methods.” **(B)** MFIs of DC-associated molecules are shown after excluding CD2^+^HLA-DR^–^ T cells. Mean ± SEM of 12 donors. Data are representative of 3 independent experiments. *P < 0.05, **P < 0.01, ***P < 0.001, ****P < 0.0001, ns, not significant. Data were analyzed with *unpaired T-test.*

To analyze the cytokine secretion profiles of DC_*Reg*_, DC_*Th*_, and MΦ_*Treg*_, each population was stimulated overnight with LPS, and selected cytokines were measured in the supernatants. LPS-stimulated DC_*Reg*_ secreted nearly 1000-fold more of the anti-inflammatory cytokine IL-10 than DC_*Th*_, but less than MΦ_*Treg*_ (Figure 3F). Conversely, DC_*Reg*_ produced significantly less IL-1β, IL-6 and TNFα than DC_*Th*_ but more than MΦ (Figure 2F). Real-time PCR analysis of FACS purified myeloid cells from these cultures indicated that the RNA expression profiles matched the cytokine secretion patterns, with DC_*Reg*_ expressing mainly IL-10 (Supplementary Figure S3 and Figure 4D) and only small amounts of inflammatory cytokines (Supplementary Figure S3).

To assess the endocytic capacity of these cells, we incubated each population with DQ-OVA, a self-quenched conjugate of OVA that emits green fluorescence after receptor-mediated internalization and proteolysis (26, 27). After a 60 min incubation, MΦ_*Treg*_ had the highest level of DQ-OVA (green fluorescence) followed by DC_*Reg*_ and DC_*Th*_ (Figure 2G; left). Furthermore, when the phagocytic activity of these cells was assessed on the basis of *E. coli* particle uptake, DC_*Reg*_ displayed strong particle uptake, second only to that of MΦ_*Treg*_ (Figure 2G; right).

Taken together, these results indicate that the phenotype, cytokine secretion profile and endocytic/phagocytic capacity of DC_*Reg*_ are distinct from those of DC_*Th*_ and MΦ_*Treg*_.

### DC_*Reg*_ Have a Unique Transcriptome

The distinctive morphology, phenotype and functions of DC_*Reg*_ prompted us to compare the gene expression profiles of DC_*Reg*_ and DC_*Th*_, MΦ_*Treg*_ and classical moDCs derived from monocytes cultured with GM-CSF and IL-4 (DC_*GM*_). Principal component analysis revealed that DC_*Th*_, DC_*Reg*_ and MΦ_*Treg*_ had distinct transcriptomes from that of DC_*GM*_ (Figure 3A). The first principal component accounted for 61% of the overall variance and clearly separated the DC_*GM*_ population from the other three populations. The DC_*Th*_, DC_*Reg*_, and MΦ_*Treg*_ separated into divergent populations based on the second principal component, which accounted for 30.7% of the variance in the dataset (Figure 3A). Next, we analyzed the 1334 differentially expressed genes (FDR adjusted *p*-values, *P* < 0.05) between DC_*Reg*,_ DC_*Th*_, MΦ_*Treg*_ and DC_*GM*_. Consistent with principal component analysis, DC_*GM*_ differed from DC_*Reg*,_ DC_*Th*_, and MΦ_*Treg*_ (Figure 3B). DC_*Reg*_ exhibited a transcriptome that was intermediate between that of DC_*Th*_ and MΦ_*Treg*_ and closer to that of DC_*Th*_ than MΦ_*Treg*_ (Figure 3B), which is consistent with their DC morphology (Figure 2B) and cell-surface phenotype (Figures 2D,E).

We further analyzed the three myeloid populations by selecting differentially expressed genes corresponding to the biological functions of antigen processing (GO 0019882) (Figure 3C), chemotaxis (GO 0006935) (Figure 3D), and endocytosis (GO 0006897) (21) (Figure 3E). Hierarchical clustering of these gene sets showed that the antigen-processing signature of DC_*Reg*_ was more closely related to that of DC_*Th*_ than MΦ_*Treg*_ (Figure 3C). Also, DCs or MΦ generated from T cell-monocyte cocultures were distinct from DC_*GM*_ in their antigen-processing signature (Figure 3C), which is consistent with their respective total transcriptome analysis (Figure 3B). By contrast, the chemotaxis (Figure 3D) and endocytosis (Figure 3E) transcriptomic pathways of DC_*Reg*_ were more similar to those of MΦ_*Treg*_ than to DC_*Th*_, and distinct from those of DC_*GM*_ (Figures 3D,E).

Recently, single-cell RNA sequencing (scRNA-seq) led to the identification of six human DC subsets (20): DC1 (CD141^+^Clec9A^+^), DC2 (CD1c^+^_A), DC3 (CD1c^+^_B), DC4 (CD1c^–^CD141^–^CD11c^+^), DC5/AS DCs (AXL^+^ SIGLEC6^+^) and DC6 (pDCs). To address whether and where DC_*Reg*_ fit in this new DC taxonomy, we used the method of Segura et al. (21) to compare the gene signatures of DC_*Reg*_, DC_*Th*_, MΦ_*Treg*_ and DC_*GM*_ with those of these six DC subsets and macrophages (MACRO) (21) (Figure 3F). To display the data, we transformed the information for each pairwise gene set enrichment analysis (GSEA) (12) into a dot whose color corresponds to the cell in which the gene signature was more represented. The dot area size is proportional to the NES (Normalized Enrichment Score), and the color intensity indicates the *p*-value (a darker, larger dot indicates a stronger enrichment). The results show that DC_*Reg*_ were relatively enriched for a DC1 (CD141^+^Clec9A^+^) signature when compared with MΦ_*Treg*_, and a DC2 (CD1c^+^_A) signature when compared with DC_*Th*_. As expected, compared with DC_*Reg*_ and DC_*Th*_, MΦ_*Treg*_ were more like conventional macrophages (MACRO) (21). Interestingly, DC_*Th*_ were also enriched for the DC6 (pDCs) signature when compared with the other three populations. MΦ_*Treg*_, DC_*Th*_, DC_*Reg*_ and DC_*GM*_ all showed variable similarity to DC1-4 and DC6 (Figure 3F), in contrast to 1,25-dihydroxyvitamin D3 (1,25(OH)2D3) induced tolerogenic DCs (VITD3DC) (12, 22) (Figure 3F). When analyzing differentially expressed genes (DEG) between tolerogenic DC_*Reg*_ and other T cell-induced DC_*Th*_ and MΦ_*Treg*_, we found that TLR4 was overexpressed in the DC_*Reg*_ (Figure 3G). Interestingly, the DC_*Reg*_ maintained their surface phenotype (Supplementary Figure S4), even when subjected to subsequent stimulation with LPS.

These results confirm that DCs differentiated from monocytes in the presence of CD4^+^ T cells are transcriptomically distinct from DCs differentiated from monocytes with GM-CSF and IL-4 (DC_*GM*_), and further, that DC_*Reg*_ are distinct from DC_*Th*_.

### Cell Contact, TGFβ, IL-10, and CTLA-4 Mediate DC_*Reg*_ Formation

To investigate whether direct contact between monocytes and Tregs or Th is required for DC_*Reg*_ differentiation, we cultured these populations on opposite sides of 0.4 μm transwell membranes, which prevent cell-cell interaction but permit diffusion of soluble molecules (Figure 4A). Separation of Tregs from monocytes and Th resulted in the induction of DC_*Th*_-like cells (Figure 4B, Transwell 5). However, when Th were separated from monocytes and Tregs, MΦ-like cells were induced (Figure 4B, Transwell 7). Monocytes cultured in transwells and separated from Th or Tregs (Figure 4B, Transwell 6&8) retained their monocyte-like phenotype. Moreover, when monocytes were cultured in transwells and separated from both Th and Tregs, the monocytes became MΦ_*Treg*_-like (Supplementary Figure S5). Thus, both Tregs and Th require direct contact with monocytes to mediate their effects on DC differentiation and the contribution of activated Th cells could not be replaced by exogenous GM-CSF and IL-4 (Supplementary Figure S5).

**FIGURE 5:**
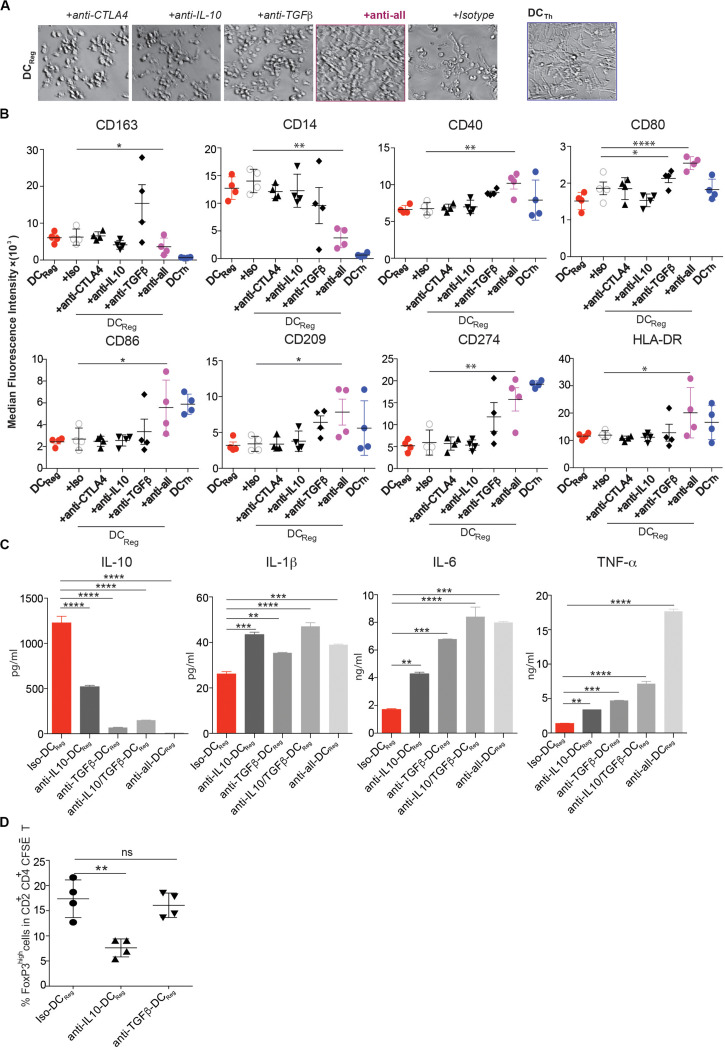
Blockade of CTLA-4, IL-10 and TGF-β prevents DC_*Reg*_ differentiation. **(A–D)** CD14^+^ monocytes were cultured for 4 days with Tregs or Th alone, or with Tregs and together at a 1:1 ratio in the presence of anti-CTLA-4 (5 μg/ml), anti-IL-10 (2 μg/ml), anti-TGFβ (2 μg/ml), or a mixture of these antibodies or isotype control antibody (5 μg/ml). **(A)** Images were captured with a bright-field DIC microscope on day 4. Original magnification 200×. **(B)** MFIs of DC-associated molecules on monocyte-derived cells are shown after excluding CD2^+^HLA-DR^–^ T cells. **(C)** On day 4, moDCs (HLA-DR^+^CD2^–^ cells) from the cultures in **(A,B)** were purified by FACS, and 200,000 cells were cultured in 200 μl containing 1 μg/ml LPS. After 16h, IL-10, IL-1β, IL-6 and TNFα levels in the cell-free supernatants were determined by Luminex. **(D)** On day 3, 1 μg/ml LPS was added to selected wells, and 16h later FACS-purified DC subsets were further cultured with MACS sorted CFSE-labeled naïve allogeneic CD4^+^ T cells. After another 6 days, CD2^+^CD4^+^ T cells were analyzed by flow cytometry. Mean ± SEM of 4 donors. *P < 0.05, **P < 0.01, ***P < 0.001, ****P < 0.0001, ns, not significant. Data were analyzed with *one-way* ANOVA, followed by Dunnett’s test for multiple comparisons.

We next sought to determine whether Treg-derived molecules that are known to impact DC function in other settings are necessary for DC_*Reg*_ differentiation (28). DC_*Reg*_ induction cultures were performed in the presence of neutralizing mAbs against CTLA-4, IL-10 or TGFβ. Each of these mAbs partially prevented DC_*Reg*_ formation, as indicated by the increased frequency of cells exhibiting a DC_*Th*_-like morphology with more extensive dendrites in these cultures compared to cultures containing isotype control antibody (Figure 5A). When all 3 neutralizing mAbs were used simultaneously (anti-all), the resultant cell morphology was comparable to that of DC_*Th*_ (Figure 5A). We also analyzed the effects of each blocking antibody individually on the expression of DC-associated surface molecules. Blocking TGFβ affected the expression of several of these molecules, but only after blocking all 3 molecules was the surface phenotype of the resulting DCs comparable to that of DC_*Th*_ (Figure 5B). These mAbs also altered surface marker expression in MΦ induction cultures (Supplementary Figure S6). Taken together, these findings suggest that TGFβ, IL-10 and CTLA-4 govern the formation of DC_*Reg*_.

To determine the impact of TGFβ, IL-10 and CTLA-4 on the acquisition of DC_*Reg*_ function, we analyzed the cytokine secretion profile and Treg-inducing potential of FACS purified DC_*Reg*_ formed under conditions in which one or more of these factors were neutralized. Neutralization of TGFβ or IL-10 increased the expression of proinflammatory cytokines IL-1β, IL-6 and TNFα by the DCs, while decreasing their expression of IL-10 (Figure 5C). Importantly, neutralization of IL-10, but not TGFβ prevented the resultant DCs from acquiring the capacity to induce Foxp3^*high*^ T cells (Figure 5D). Thus, IL-10 is required for the formation of functionally active DC_*Reg*_, while functionally active DC_*Reg*_ utilize TGFβ to induce new FoxP3^+^ Tregs.

### Human Colorectal Cancers (CRC) Contain DC_*Reg*_, and T Cells From the Same Tumors Induce DC_*Reg*_ Formation From Monocytes

**FIGURE 6:**
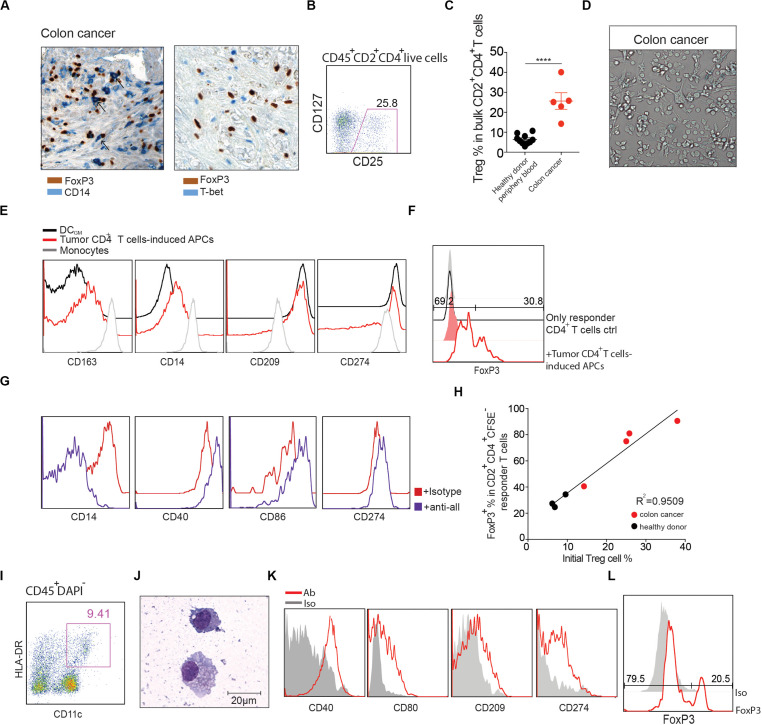
CRC CD4^+^ T cells induce DC_*Reg*_ formation. **(A)** Left: representative human colon cancer tissue stained with antibodies against FoxP3 (brown) and CD14 (blue). Black arrows represent potential monocyte-Treg interactions. Right: representative human colon cancer tissue stained with antibodies against FoxP3 (brown) and T-bet (blue). Magnification 400×. **(B)** Representative flow cytometry analysis of CD25^+^CD127^*low*^ Tregs in CD45^+^CD2^+^ CD4^+^ T cells from colon cancer sample. **(C)** Comparison of the CD25^+^CD127^*low*^ Treg percentages from healthy donor peripheral blood and colon cancer samples. Mean ± SEM. ****P < 0.0001, unpaired *T* test. **(D,E)** Lin^–^CD45^+^CD2^+^CD4^+^ T cells were purified from human CRC and co-cultured with peripheral blood CD14^+^ monocytes at a 1:10 ratio in the presence of anti-CD3 mAb for 4 days. **(D)** Images were captured with a bright-field DIC microscope on day 4. Original magnification 200×. **(E)** Flow cytometry analysis of the resultant antigen presenting cells (HLA-DR^+^CD2^–^) (red line) compared to monocytes cultured alone (gray line) or with GM-CSF and IL-4 (black line). **(F)** CD11c^+^HLA^–^DR^+^CD2^–^ cells were purified via FACS and stimulated for 16 h with LPS. Cells were washed extensively and cultured with CFSE-labeled allogeneic naïve CD4^+^ T cells in a MLR. The percentage of FoxP3^+^ cells in CD2^+^CD4^+^CFSE^–^ cells was determined 6 days later (red line). Naïve CD4^+^ T cells cultured alone (black line) are shown as controls. Shaded histograms represent isotype controls. Data are representative of 3 independent experiments. **(G)** In the presence of anti-CD3 mAb, CD14^+^ monocytes were cultured with bulk CD45^+^CD2^+^CD4^+^ T cells sorted from CRC, in the presence of a mixture of anti-CTLA-4 (5 μg/ml), anti-IL-10 (2 μg/ml), anti-TGFβ (2 μg/ml), or isotype control antibody (5 μg/ml). At day 4, cells in culture were analyzed by flow cytometry for the indicated surface markers. **(H)** CD14^+^ monocytes were cultured with bulk CD45^+^CD2^+^CD4^+^ T cells sorted from CRC (red) or healthy donor peripheral blood (black). CD25^+^CD127^*low*^ Treg percentages were analyzed before coculture. The graph shows the correlation between the percentage of Tregs in CD2^+^CD4^+^ T cells prior to their initial culture with monocytes (*X* axis) and the percentage of CFSE^–^FoxP3^+^ T cells among the responder CD4^+^ T cells cultured with the DCs generated in the initial culture (*Y* axis). Linear regression was determined by prism. *R*^2^ = 0.9509. *P* = 0.0002. **(I)** Representative flow cytometry analysis of human CRC DCs. DAPI^–^CD45^+^ cells were further gated with CD11c, HLA-DR. **(J)** Cytospin of freshly FACS-sorted CD11c^+^HLA-DR^+^ DCs with Grunwald-Giemsa staining from CRC. Scale bar as indicated. **(K)** Flow cytometry analysis of CD40, CD80, CD86 and CD274 expression on gated CD11c^+^HLA-DR^+^ DCs. Red line: Ab staining. Shaded histograms represent isotype controls. **(L)** CD45^+^CD11c^+^HLA^–^DR^+^ DCs from CRC were sorted by FACS and cultured with CFSE-labeled naïve allogeneic CD4^+^ T cells. The percentage of FoxP3^+^ cells in CD2^+^CD4^+^CFSE^–^ cells was determined 6 days later.

Since Tregs (29) and monocytes (30) are often abundant in human tumors, including CRC, where their phenotypes and functions are often altered (31), we tested the hypothesis that interactions between these cells might result in the generation of DC_*Reg*_. We utilized immunohistochemistry to identify Tregs and CD14^+^ cells in human CRC, and found that these cells were often in close apposition to one another (Figure 6A; left), and conventional Th (T-bet^+^) cells were also identified in the same regions (Figure 6A; right). Flow cytometric analysis of single cell suspensions prepared from 5 CRC patients indicated that CD25^+^CD127^*low*^ Tregs comprised approximately 25% of total CD45^+^CD2^+^CD4^+^ T cells (Figure 6B), which is similar to that reported previously for CRC (20) and much higher than in surrounding uninvolved tissue (Supplementary Figure S7) or healthy donor peripheral blood (Figure 6C).

Given the relative ratios of monocytes, Tregs and Th in these tumors, we hypothesized that the conditions would favor formation of DC_*Reg*_. As a first step to investigate this possibility, we evaluated the ability of unfractionated tumor CD4^+^ T cells to induce DC_*Reg*_ from monocytes, *in vitro*. Thus, FACS purified CD4^+^ T cells from CRC tissues were cultured with peripheral blood monocytes at a 1:10 ratio in the presence of an agonistic anti-CD3 monoclonal antibody (mAb). HLA-DR^+^CD2^–^ cells with DC morphology appeared within 4 days of culture initiation (Figure 6D). Consistent with the phenotype of DC_*Reg*_ (Figures 2D,E), these cells expressed characteristic DC markers such as CD209, CD274, CD40, and CD86, and also expressed monocyte-defining markers including CD14 and CD163 (Figure 6E). When these DCs were cultured for 1 week with allogeneic naïve CD4^+^ T cells (> 99% purity; Supplementary Figure S2), > 30% of the responder T cells expressed FoxP3 (Figure 6F). Moreover, in accord with our data from healthy donors (Figure 5B), neutralization of TGFβ, IL-10 and CTLA-4 inhibited DC_*Reg*_ formation (Figure 6G).

Given that CD4^+^ T cells isolated from tumor tissue were enriched for Tregs, we hypothesized that the ratio of Tregs to Th in tumors would correlate with the capacity of the resultant DCs to induce FoxP3^+^ Tregs. To investigate this possibility, we quantified the percentage of Tregs in CD4^+^ T cells in tumor specimens and co-cultured total tumor CD4^+^ T cells with peripheral blood monocytes. Subsequently, we analyzed the capacity of the resultant DCs to elicit FoxP3^+^ Treg differentiation from naïve T cells stimulated in an MLR. The results indicate that the percentage of tumoral Tregs strongly correlated with the capacity of the ensuing DCs to induce FoxP3 expression, *R*^2^ = 0.9509 (Figure 6H).

Studies of the same tumor specimens revealed that the majority (∼80%) of tumor-associated DCs (TADCs), defined as CD45^+^CD11c^+^HLA-DR^+^ cells, expressed CD14, which indicates their monocytic origin and likely inclusion among the CD14^+^ cells associated with Tregs (Figures 6I,J). Staining for additional DC surface markers showed that they expressed CD40, CD80, CD86 and CD274 (Figure 6K). When these CRC mo-DCs were incubated with naïve CFSE-labeled naïve allogeneic CD4^+^ T cells (> 99% purity; Supplementary Figure S2) in a mixed leukocyte reaction (MLR), a high proportion (> 20%) of the responder T cells expressed FoxP3 (Figure 6L). In summary, our findings suggest that not only are all of the cellular requirements for generating DC_*Reg*_ present in these tumors, but also DC_*Reg*_ similar to those induced from monocytes *in vitro* are present *in situ* in the same tumors.

## Discussion

Human monocytes can be induced to differentiate into tolerogenic DCs upon exposure to growth factors, cytokines, or pharmacological agents, *in vitro* (32). However, if and how human Tregs impact DC differentiation from monocytes has not been described. Here, we have demonstrated the ability of natural Tregs to promote the formation of DC_*Reg*_ directly from monocytes, and thereby reinforce an immunosuppressive environment through the induction of increased numbers of induced FoxP3^+^ Tregs. Our study reveals a novel mechanism whereby Tregs and Th collaborate in the induction of human regulatory DCs, thereby inducing immunosuppression and potentially contributing to “infectious tolerance” (33, 34).

By studying the effects of Tregs and Th, separately and in combination, on monocytes, we were able to analyze the mechanism responsible for DC_*Reg*_ formation. In agreement with our prior findings, cultures containing only activated Th and monocytes led to the formation of DC_*Th*_ that secreted substantial amounts of IL-6 and TNFα and little or no IL-10 (8). Consistent with a previous report, monocytes cultured with Tregs alone differentiated into cells that are indistinguishable from macrophages (35). However, when equal numbers of Tregs and Th were added at the initiation of these cultures, the resultant morphology, surface phenotype and functionality of the monocyte–derived cells (DC_*Reg*_) were distinct. Based on their large nuclei, dense cytoplasm, presence of surface dendrites and low expression of the MΦ marker MerTK (36), these monocyte-derived cells are more similar to DCs than MΦ. However, their surface phenotype and phagocytic activity indicate that they have features of both DC_*Th*_ and MΦ. Their secretion of IL-10 but not proinflammatory cytokines, combined with their ability to induce the formation of FoxP3^+^ Tregs from naïve T cells via TGFβ, suggests that their major function is to suppress the immune response.

Monocytes are highly mobile and plastic and, therefore, ideally equipped to respond to inflammatory or immunoregulatory signals. Our studies show that the signals present during monocyte differentiation determined not only whether DCs or MΦ develop, but also the functions of the differentiated cells. DC_*Reg*_ differentiated from monocytes were morphologically, functionally and transcriptomically distinct from other moDCs. DC_*Reg*_ were also distinct from the previously described Treg-treated DC (Treg-DC) in their phagocytic capacity and cytokine secretion profile, although both can induce FoxP3^+^ Tregs (37). Although DC_*Reg*_ expressed comparatively higher levels of TLR4 compared with DC_*Th*_ and MΦ_*Treg*_, their phenotype remained relatively stable upon LPS stimulation. Thus, DC_*Reg*_ appear to be differentiated DCs that function mainly to induce Foxp3^+^ Tregs from CD4^+^ naïve T cells. These results are consistent with our previous finding that monocytes induced to differentiate into DC_*Th*_ by different Th subsets develop into relatively stable DC populations that promote the polarization of the same Th subsets responsible for DC differentiation (8).

Tregs promoted the generation of DC_*Reg*_ from monocytes, but they did so only in the presence of activated Th, and only if all 3 cell types were in direct contact. Interestingly, IL-10 but not TGFβ was required for the formation of functionally active DC_*Reg*_, while TGFβ but not IL-10 was required for FoxP3^+^ T cell induction by DC_*Reg*_. MΦ derived from culturing monocytes with Tregs alone secreted large amounts of IL-10 (35). However, unlike DC_*Reg*_, they failed to induce FoxP3^+^ Tregs, indicating that the formation of DC_*Reg*_ requires DC differentiation signals from Th, in addition to polarizing signals from Tregs.

The FoxP3^+^ Tregs induced by DC_*Reg*_ are, by definition, inducible Tregs (iTregs) as opposed to thymus-derived natural Tregs. Whereas expression of FoxP3 is considered a definitive marker of murine Tregs, this is not the case in humans. Indeed, one study showed that activation of human T cells with anti-CD3 and anti-CD28 in the presence of TGFβ resulted in the generation of FoxP3^+^ T cells that were not suppressive and produced high levels of effector cytokines (38). Our findings differ from this study in that DC_*Reg*_ induced the development of FoxP3^+^ T cells that are functionally suppressive. Interestingly, although both IL-10 and TGFβ contributed to the morphology and surface phenotype of DC_*Reg*_ induced by Tregs, IL-10 but not TGFβ was required for the formation of functionally active DC_*Reg*_. On the other hand, TGFβ but not IL-10 was required for FoxP3^+^ T cell induction by DC_*Reg*_.

We also found DC_*Reg*_-like cells in CRC specimens, and they are likely present in a wide range of human tumors given the high frequency of Tregs, monocytes and DCs in many tumor types. Their expression of the monocyte/macrophage related markers CD64 and MerTK on CD11b^+^ TADCs described by Broz et al. also suggests their mixed ontogeny (39). Indeed, a significant portion of the myeloid cells in these and other tumors are macrophages. Comprehensive gene expression profiling at single-cell resolution will provide a more accurate indication of the frequency and location of DC_*Reg*_ in tumors relative to other myeloid cells. Nonetheless, in addition to DC_*Reg*_, the CRC specimens contained a high proportion of Tregs relative to Th, and when monocytes were cultured with total CD4^+^ T cells from these tumors, they differentiated into phenotypically and functionally similar DC_*Reg*_. As conditions favoring the development of DC_*Reg*_ are present in many tumors, these cells are likely important contributors to tumor immune tolerance across a broad range of tumor types. Moreover, we speculate that regulatory DCs similar to those described here are likely present in tissues, in addition to tumors, that contain an abundance of myeloid cells along with Th and Tregs (29, 40).

## Data Availability Statement

The datasets presented in this study can be found in online repositories. The names of the repository/repositories and accession number(s) can be found below: https://www.ncbi.nlm.nih.gov/, GSE148114.

## Ethics Statement

The studies involving human participants were reviewed and approved by Stanford Research Compliance Office under Stanford IRB protocol #6304. Written informed consent for participation was not required for this study in accordance with the national legislation and the institutional requirements.

## Author Contributions

XZ, MA, and EE conceived the study. XZ designed and performed the research, analyzed the data, made figures, and wrote the manuscript. MA helped in designing the experiments and edited the manuscript. PZ analyzed the microarray data. TP helped in analyzing microarray data. MA, HZ, YC, DW, and E-SK performed the experiments. LT, NW, and OC performed the FACS sorting. SS helped in designing and interpreted the experiments. EE supervised the study and wrote the manuscript. All authors contributed to the article and approved the submitted version.

## Conflict of Interest

The authors declare that the research was conducted in the absence of any commercial or financial relationships that could be construed as a potential conflict of interest.

## Footnotes

1 https://www.r-project.org/
