# Supplementary material for: Human Regulatory Dendritic Cells Develop From Monocytes in Response to Signals From Regulatory and Helper T Cells
Source: Front Immunol. 2020 Aug 18;11:1982. doi: 10.3389/fimmu.2020.01982 (PMC7461788; doi:10.3389/fimmu.2020.01982)
Supplement: Supplementary file 1 [file Data_Sheet_1.docx]

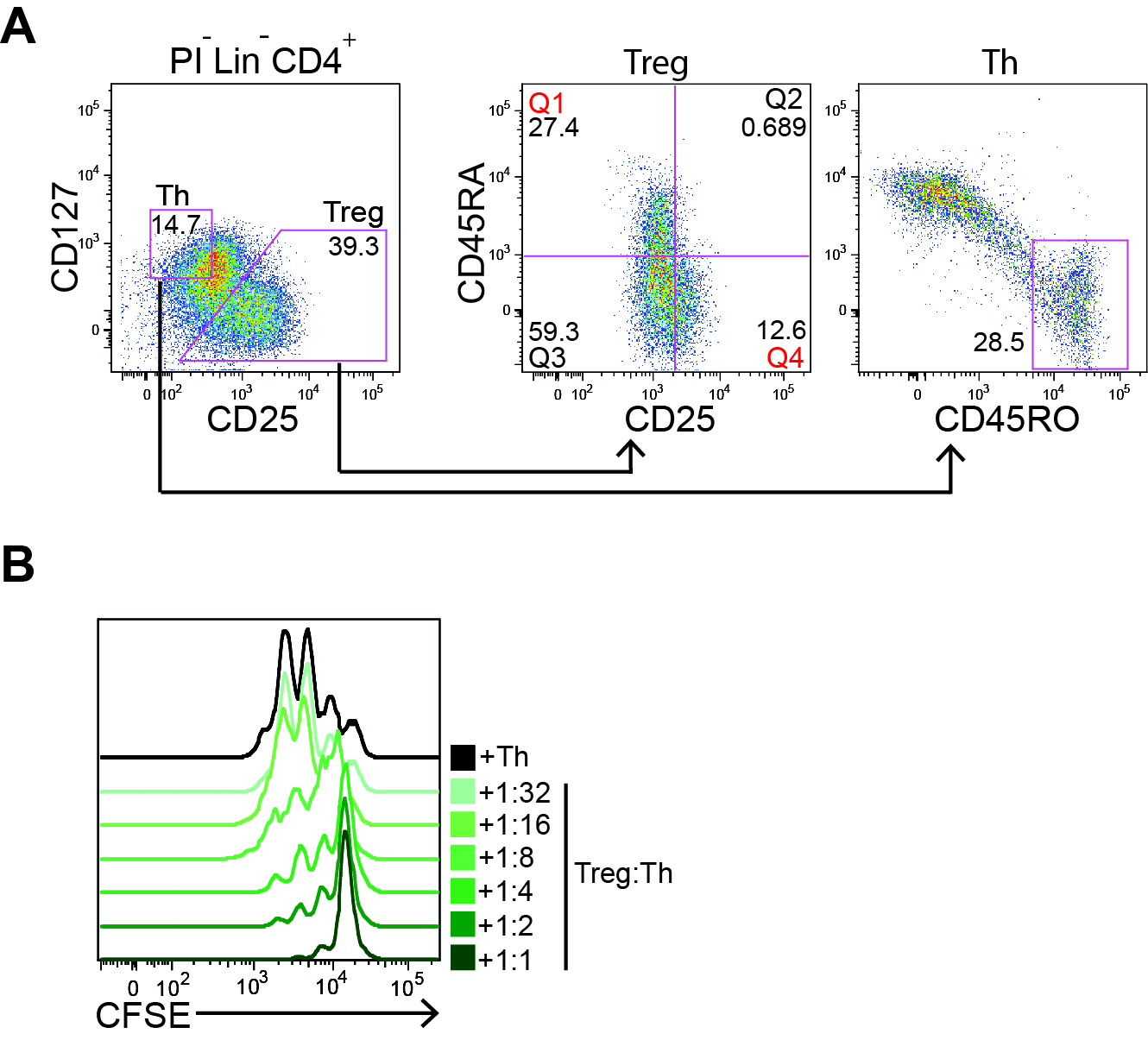


**Fig S1.** (A) Sorting strategy for human Tregs and memory Th. Tregs (Q) and memory Th were isolated from PBMCs according to their expression of CD127, CD25, CD45RA and CD45RO. Numbers adjacent to gated areas indicate the percentage of cells before sorting. (B) Flow cytometry analysis of the proliferation status of CFSE-labeled Th in the coculture of Th with different doses of Tregs under anti-CD3 mAb (50ng/ml) stimulation. Data are representative of at least 3 independent experiments.

**
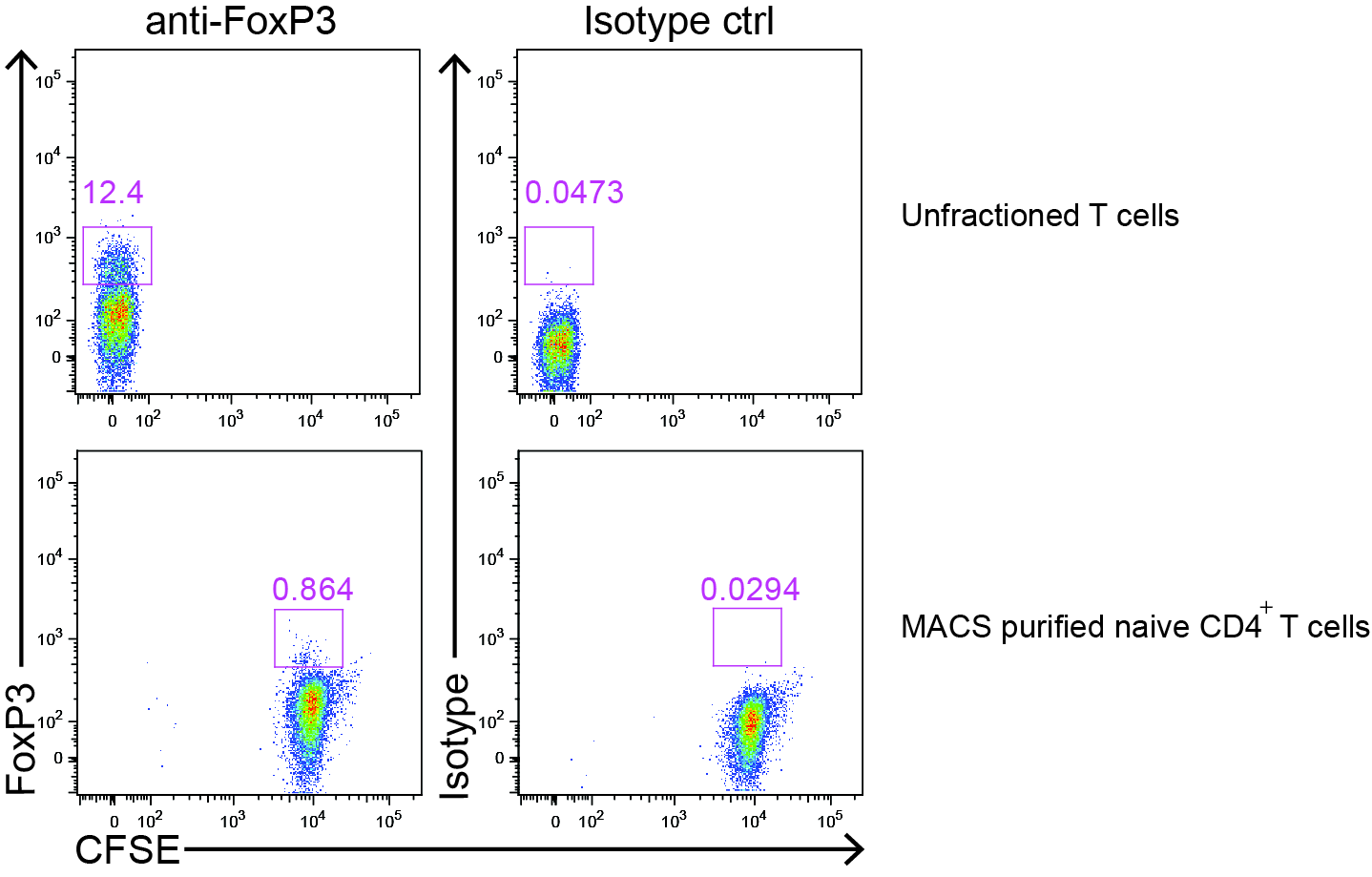
**

**Fig S2. FoxP3 expression of MACS-sorted naïve CD4^+^ T cells**. T cells were isolated from PBMCs obtained from healthy blood donors by density gradient centrifugation, and naïve CD4^+^ T cells were purified by MACS and labeled with CFSE. Cells were stained for FoxP3 expression. Left: FoxP3 vs. CFSE on HLA-DR^-^CD2^+^CD4^+^ cells; right: isotype control staining. Numbers adjacent to gated areas indicate percentage of gated cells.

**
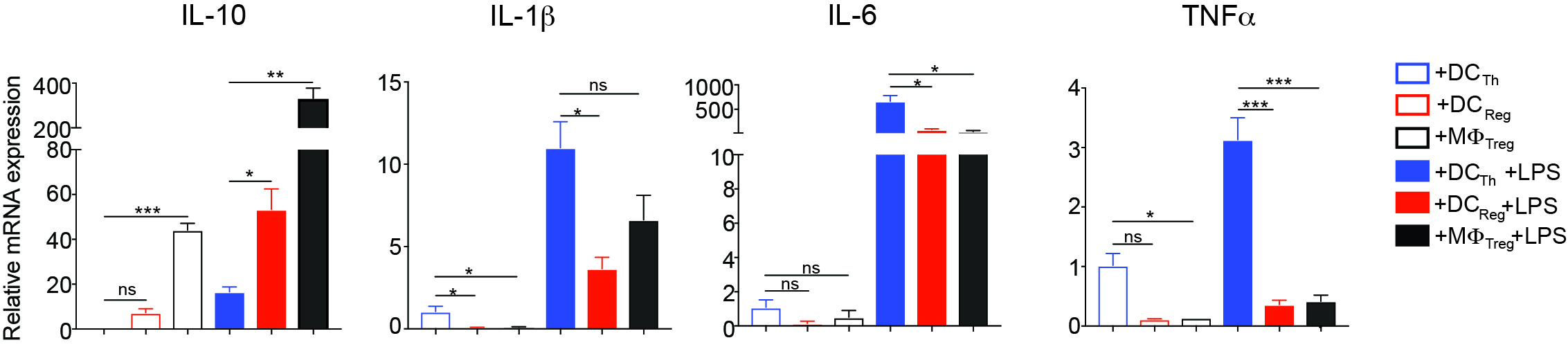
**

**Fig S3. Cytokine expression levels of monocyte-derived myeloid cells by quantitative real-time RT-PCR**. CD14^+^ monocytes were cultured with Treg (MΦ_Treg_) or memory Th (DC_Th_) alone, or with both Treg and Th at 1:1 (DC_1:1_) ratio. Where indicated, 1µg/ml LPS was added on day 3. 16 hr later the cultures were harvested and HLA-DR^+^ CD2^-^ cells were sorted by flow cytometry. The expression levels of IL-10, IL-1β, IL-6 and TNFα have been normalized with the average expression of the endogenous control β-actin. Data are representative of at least two independent experiments. *P <0.05, **P <0.01, ***P <0.001, ****P <0.0001. Data were analyzed with one-way ANOVA, followed by Dunnett’s test for multiple comparisons.


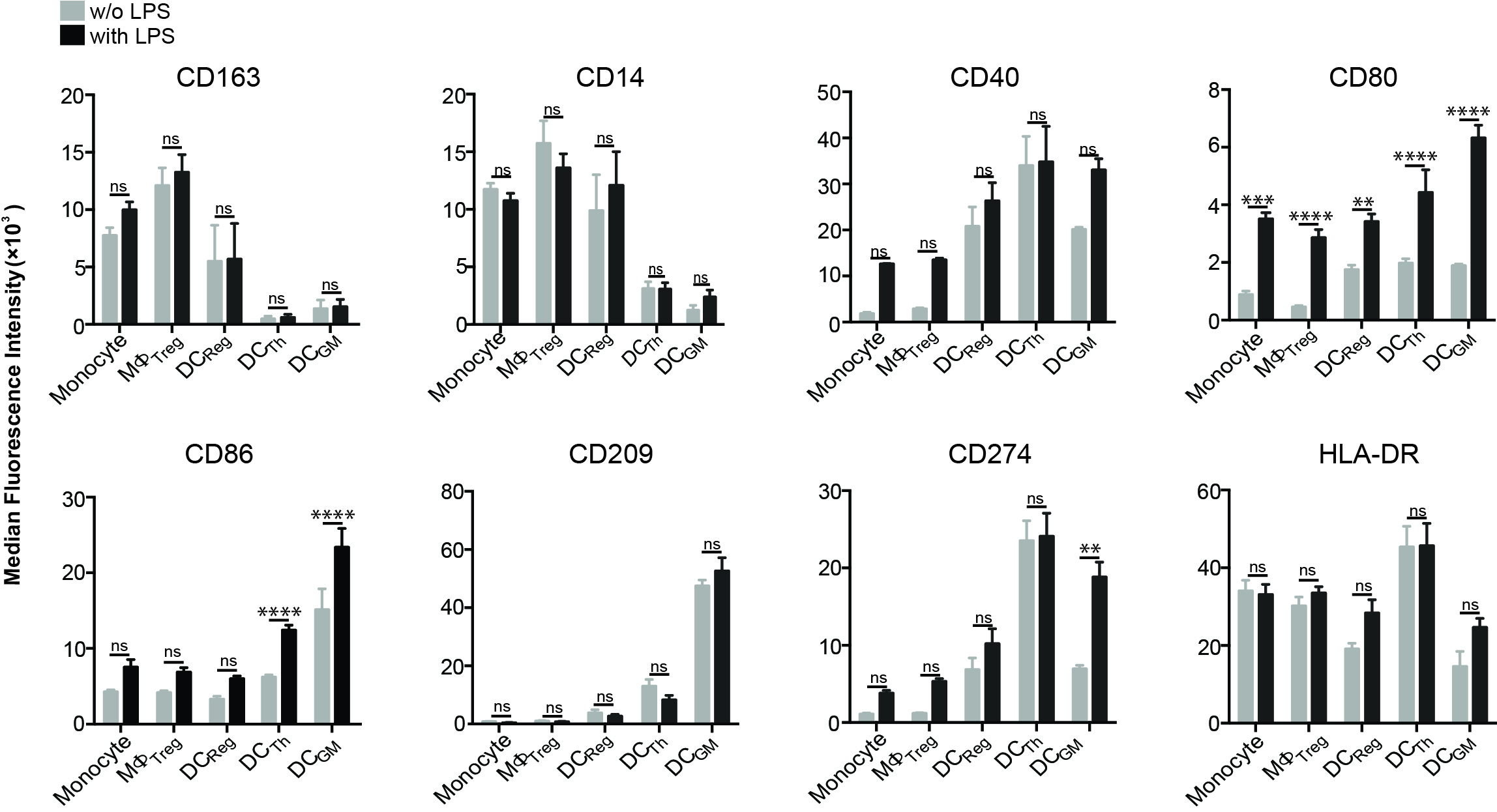


**Fig S4. LPS has only modest effects on the surface phenotype of DC_Reg_**. In the presence of anti-CD3 mAb (50ng/ml), CD14^+^ monocytes were cultured with allogenic Treg or memory Th alone, or with Treg and Th together at 1:1 ratio. After 3 days, cells were cultured with or without 1µg/ml LPS for 16 hours. On day 4, cells were harvested for analysis. Median fluorescence intensities (MFIs) of DC-associated molecules are shown after excluding CD2^+^HLA-DR^-^ T cells. Mean±SEM of 5 donors from one representative of three independent experiments. *P <0.05, **P <0.01, ***P <0.001, ****P <0.0001. Data were analyzed with two-way ANOVA, followed by Sidak's post hoc test for multiple comparisons*.*


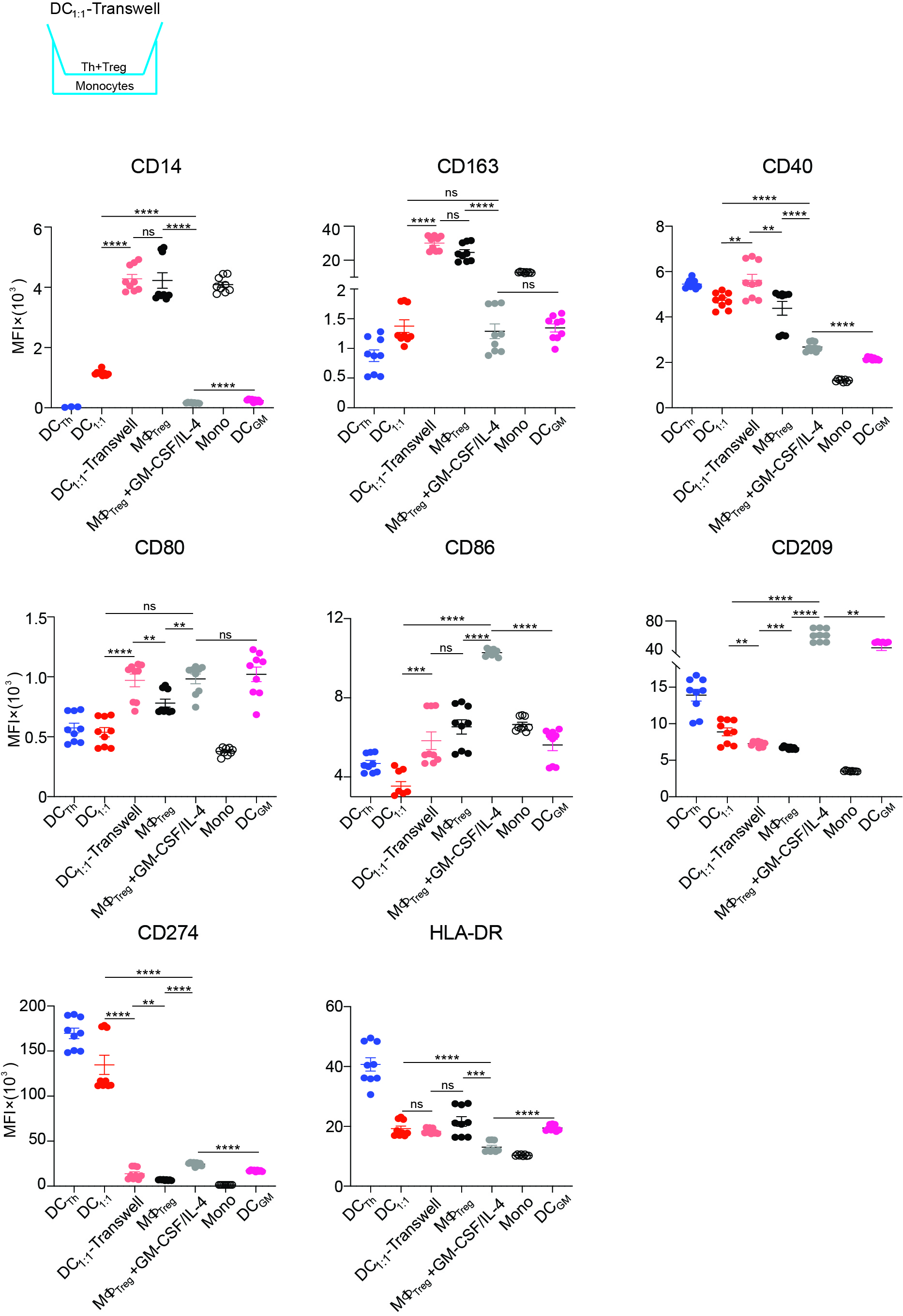


**Fig S5. Soluble factors cannot substitute for Tregs and Th cells in the generation of DC_Reg_.** Th and Tregs were separated with monocytes as indicated (DC_1:1_-Transwell). Monocytes were cultured with Tregs in the presence of GM-CSF and IL-4 (MΦ_Treg_+GM-CSF/IL-4). Other conditions are as described in Figure 2 and Figure 3. DC_GM_ were derived from monocytes cultured with GM-CSF and IL-4. Data were analyzed with an unpaired T-test and are representative of 3 independent experiments. *P <0.05, **P <0.01, ***P <0.001, ****P <0.0001.


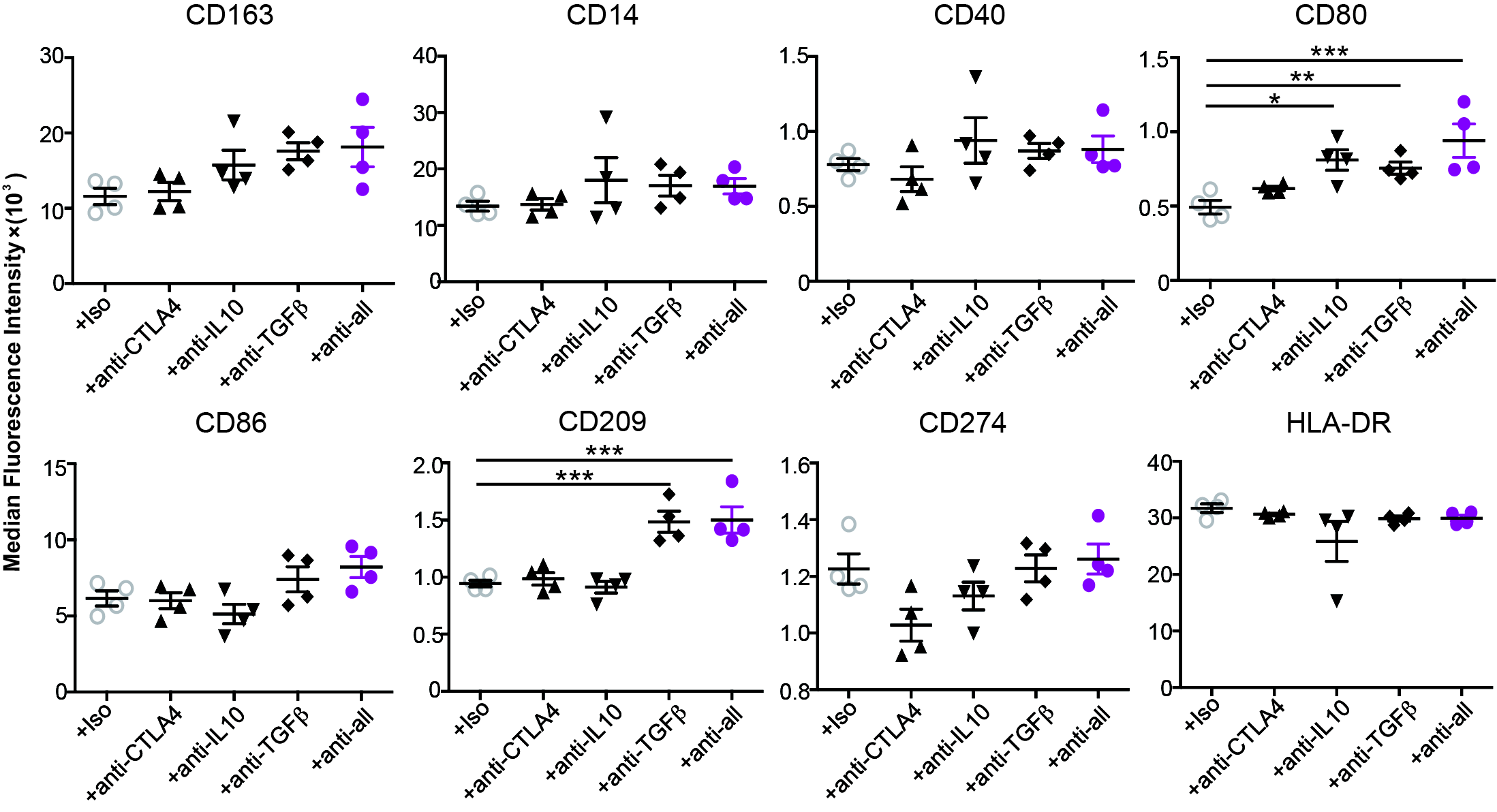


**Fig S6.** **Effects of neutralizing antibodies against CTLA-4, IL-10 and TGF-β on MΦ_Treg_ differentiation**. CD14^+^ monocytes were cultured for 4 days with Tregs alone in the presence of different blocking antibodies (anti-CTLA-4: 5µg/ml; anti-IL-10: 2µg/ml; anti-TGF-β: 2µg/ml) or isotype control antibody (5µg/ml). MFIs of DC surface molecules as analyzed by flow cytometry are shown after excluding CD2^+^HLA-DR^-^ T cells. Mean±SEM of 4 donors. Data are representative of 3 independent experiments. *P <0.05, **P <0.01, ***P <0.001, ****P <0.0001. Data were analyzed with one-way ANOVA, followed by Dunnett’s test for multiple comparisons.


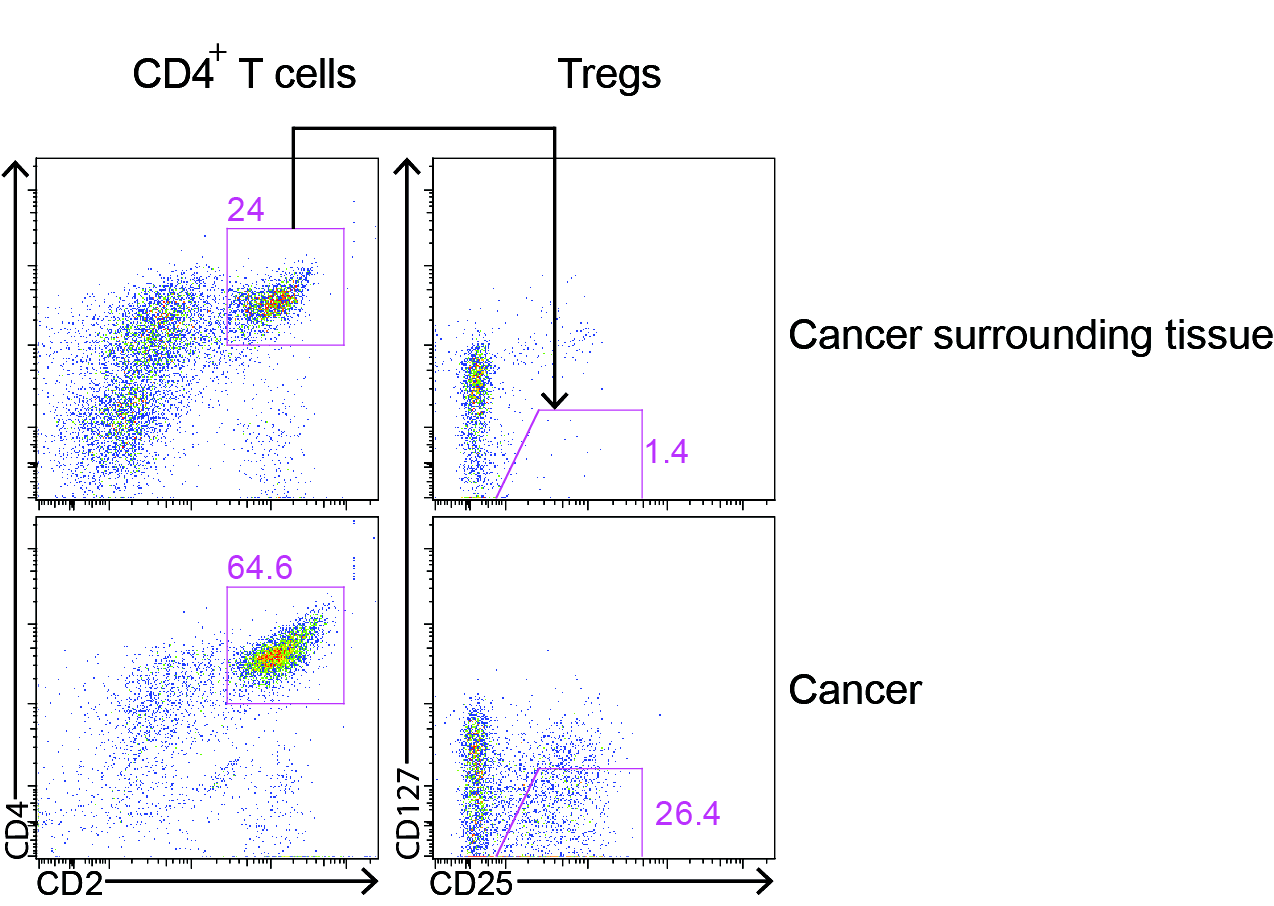


**Fig S7. Flow cytometry analysis of Tregs in human colorectal cancers and surrounding healthy tissues.** CD4^+^ T cells were gated as CD2^+^CD4^+^ (left) from MACS-purified CD45^+^ cells, and Tregs were further gated as CD25^+^CD127^low^ (right). Numbers adjacent to gated areas indicate percentage of gated cells. Data are representative of 4 experiments.

**Supplemental Experimental Procedures**

***Transmission electron microscopy***

Samples were fixed in Karnovsky’s fixative: 2% Glutaraldehyde (EMS Cat# 16000) and 4% Formaldehyde (EMS Cat# 15700) in 0.1M Sodium Cacodylate (EMS Cat# 12300) pH 7.4 for 1 hr, chilled and sent to Stanford’s CSIF (The Cell Sciences Imaging Facility) on ice. They were then allowed to warm to room temperature (RT) in cold 1% osmium tetroxide (EMS Cat# 19100) for 1 hr in a hood, washed 3X with ultrapure water, then stained en bloc overnight in 1% Uranyl Acetate at 4^o^C while rotating. Samples were then dehydrated in a series of ethanol washes for 30 min each @ 4°C, beginning at 50% and then proceeding to 70%, 95% and 100% ethanol 2X, followed by Propylene Oxide (PO) for 15 min. Samples were infiltrated with EMbed-812 resin (EMS Cat#14120) mixed 1:2, 1:1, and 2:1 with PO for 2 hr each before leaving samples rotating in 2:1 resin to PO overnight at RT. The samples were then placed in EMbed-812 for 2-4 hr, before being transferred into molds with fresh resin and warmed overnight at 65°C. 75 and 90 nm sections were applied to formvar/Carbon-coated slot Cu grids, stained for 30 sec in 3.5% Uranyl Acetate in 50% Acetone followed by staining in 0.2% lead citrate for 3 minutes. The grids were analyzed in a JEOL JEM-1400 120kV and photos were taken using a Gatan Orius 4k X 4k digital camera.

***Endocytosis and phagocytosis***

Cells were harvested from monocyte-T cell cocultures on day 4 and incubated with 1mg/ml DQ-OVA (Life Technologies) at 37°C for 60min. Phagocytosis was assayed by incubating cells harvested from monocyte-T cell cocultures on day 4 with pHrodo™ Red E. coli BioParticles® Phagocytosis Kit (Life Technologies) for 60 min at 37°C. Cells were analyzed by flow cytometry.

***CFSE labeling***

Purified Th or naïve CD4^+^ T cells were suspended in pre-warmed PBS with 0.1% human serum at 10^7^ cells/ml. An equal volume of 2µM CFSE was added to yield a final concentration of 1µM CFSE. Cells were incubated at room temperature for 5 min prior to stopping the reaction with 10 ml ice-cold RPMI-1640 supplemented with 10% human serum.

***RNA isolation and quantitative RT-PCR***

HLA-DR^+^CD2^-^ myeloid cells were purified from their respective cocultures by FACS on day 3. RNA was extracted with an RNeasy Micro Kit (Qiagen) and cDNA was subsequently synthesized with SuperScript II Reverse Transcriptase (Life Technologies) with random hexamer primers (Life Technologies). Quantitative RT-PCR was performed with a Taqman Fast Advanced Mastermix, Taqman gene expression and ABI QuantStudio 6 Flex. The cycling threshold (Ct) of gene transcripts for each cell population was determined by RT-PCR and normalized to the Ct of *β-actin* for calculation of the ΔCt value. Relative gene expression levels were determined in triplicate and calculated using the 2^−ΔΔCt^.
